# Modality-specificity of Selective Attention Networks

**DOI:** 10.3389/fpsyg.2015.01826

**Published:** 2015-11-25

**Authors:** Hannah J. Stewart, Sygal Amitay

**Affiliations:** Medical Research Council Institute of Hearing ResearchNottingham, UK

**Keywords:** selective attention, auditory attention, visual attention, orienting, conflict resolution, dual-pathway

## Abstract

**Objective:** To establish the modality specificity and generality of selective attention networks.

**Method:** Forty-eight young adults completed a battery of four auditory and visual selective attention tests based upon the Attention Network framework: the visual and auditory Attention Network Tests (vANT, aANT), the Test of Everyday Attention (TEA), and the Test of Attention in Listening (TAiL). These provided independent measures for auditory and visual alerting, orienting, and conflict resolution networks. The measures were subjected to an exploratory factor analysis to assess underlying attention constructs.

**Results:** The analysis yielded a four-component solution. The first component comprised of a range of measures from the TEA and was labeled “general attention.” The third component was labeled “auditory attention,” as it only contained measures from the TAiL using pitch as the attended stimulus feature. The second and fourth components were labeled as “spatial orienting” and “spatial conflict,” respectively—they were comprised of *orienting* and *conflict resolution* measures from the vANT, aANT, and TAiL attend-location task—all tasks based upon spatial judgments (e.g., the direction of a target arrow or sound location).

**Conclusions:** These results do not support our *a-priori* hypothesis that attention networks are either modality specific or supramodal. Auditory attention separated into selectively attending to spatial and non-spatial features, with the auditory spatial attention loading onto the same factor as visual spatial attention, suggesting spatial attention is supramodal. However, since our study did not include a non-spatial measure of visual attention, further research will be required to ascertain whether non-spatial attention is modality-specific.

## Introduction

The ability to selectively attend to the constantly changing stream of sensory information is a vital skill due to our limited perceptual resources (see review in Lee and Choo, 2011). The Attention Network framework proposed by Posner and Petersen (1990; updated in Petersen and Posner, 2012) divides attentional control into three separable networks: alerting, increasing arousal levels to better process new stimuli; orienting, selecting objects, or object features; and executive control, which allows resolution of conflicts to achieve a behavioral aim. Whilst both alerting and executive control are thought to be supramodal (Fernandez-Duque and Posner, 1997; Roberts and Hall, 2008), orienting has been argued to be modality-specific (Roberts et al., 2006; Spagna et al., 2015). The purpose of the current study was to assess whether attentional control can be separated into supramodal and modality-specific functions, using a comparison of auditory and visual tests of selective attention based on the Attention Network framework.

Alerting, detecting sudden, or novel stimuli by continuously monitoring the environment (Posner, 1978), is measured as the advantage to processing speed conferred by knowing exactly when a stimulus will appear, usually through presenting a temporal cue preceding the target stimulus. In the real world, objects have both visual and auditory “features,” and detecting the appearance of an object can be based on any of its features, regardless of their modality. It is therefore more efficient to monitor the environment in a supramodal fashion rather than integrating modality-specific perceptual streams. Imaging studies support the modality-generality of alerting: the same midbrain activation in the reticular formation and thalamus has been associated with continuous monitoring for both visual and somatosensory stimuli (Kinomura et al., 1996), and right-hemisphere lateral frontal cortex, anterior cingulate, inferior temporal, and thalamus were activated when utilizing auditory and visual temporal cues (Sturm and Willmes, 2001; Roberts and Hall, 2008). On the other hand, both Roberts et al. (2006) and Spagna et al. (2015) argued alerting is modality specific based on lack of correlation between behavioral auditory and visual alerting measures.

Orienting is the ability to select specific object features relevant to the behavioral goal while avoiding distraction by irrelevant features. In both vision (e.g., Fan et al., 2002) and audition (e.g., Roberts et al., 2006) it is usually measured as the advantage given by cueing the target location (see Spagna et al., 2015). However, orienting does not necessarily need to be to a specific location, as a cue can orient to non-spatial object features, such as color (Lamers et al., 2010) or pitch (Zhang et al., 2012). The orienting network may therefore depend on the modality of the object feature to be selected, and in that sense be modality-specific. This suggestion is supported by studies showing that attention can be concurrently oriented to different locations in different modalities (Spence and Driver, 1996; Spence, 2001).

Conflict resolution is one of the functions of the executive control network, measuring the ability to respond correctly to the task-relevant object features in the face of conflicting information, typically from irrelevant features. Regardless of the modality of the conflicting features, the process of resolving it is considered to be supramodal (Donohue et al., 2012; Spagna et al., 2015). A comparison of different conflict paradigms based on visual color and auditory pitch showed similar activation patterns in anterior cingulate and bilateral inferior frontal gyrus, insula, and parietal lobe (Roberts and Hall, 2008), supporting the supramodal nature of the conflict resolution network.

A variety of attention tests have utilized different stimulus features and cues to assess Posner and Petersen's (1990) alerting, orienting, and conflict resolution networks in the visual and auditory domains. The most widely used are the visual Attention Network Test (vANT; Fan et al., 2002) which uses non-verbal temporal and spatial cues, and the ecologically valid multi-modal Test of Everyday Attention (TEA; Robertson et al., 1994) which relies on task instructions but uses non-verbal stimuli. An auditory equivalent to the vANT, the auditory Attention Network Test (aANT; Roberts et al., 2006) uses temporal and spatial cues but is dependent on verbal processing. The recently developed Test of Attention in Listening (TAiL; Zhang et al., 2012) utilizes non-verbal stimuli. These tests were chosen for this study to systematically assess all three selective attention networks separately in each modality. Since the study of visual attention is much more advanced than auditory attention, this study will increase our understanding of auditory attention and the relationship between auditory and visual attention.

The current study aimed to validate Posner and Petersen's (1990) triad of constructs and test their modality specificity. However, rather than assuming that the four tests tap into these same underlying constructs we subjected the measures to an exploratory factor analysis. We reasoned that if these constructs are common across the tests, reducing the dimensionality using principal components analysis should result in these constructs re-emerging. Moreover, whereas factors that capture supramodal constructs should incorporate both auditory and visual measures, separate factors should emerge for modality-specific constructs incorporating modality-specific measures.

## Methods

### Participants

Forty-eight participants aged 19–37 (*M* = 24.2 years, *SD* = 4.8 years, 30 females and 18 males) were recruited through poster advertisements placed in the University of Nottingham. All participants had normal hearing (pure tone thresholds below or equal to 20 dB HL bilaterally at octave frequencies between 250 and 8000 Hz in accordance with the British Society of Audiology, 2011). All procedures were approved by the NHS Research Ethics Committee 1 East Midlands—Nottingham. Informed written consent was given by each participant prior to the experiment, and they were paid an inconvenience allowance.

### Apparatus

Participants were tested individually in a sound-attenuated booth. All tests, except for the TEA, were fully automated and presented on a PC, with a 15-inch flat-screen monitor placed 65 cm in front of the participant. Auditory stimuli were generated by MATLAB 2008a (MATLAB, 2008) using PsychoPhysics Toolbox v3.0.9, an ASIO driver controlled custom sound card, and presented through Sennheiser HD 25-II headphones. Visual stimuli (and feedback) were also presented through Matlab. Participants responded using a horizontally placed custom-made three-choice button box. The TEA was completed with the experimenter in the sound-attenuated chamber, with the CD-recorded stimuli presented through laptop speakers.

### Stimuli and procedure

The four tests of selective attention (see below) were administered in a single testing session lasting approximately 2 h, including rest breaks between individual tests. A random number generator was used to assign an initial order to the four attention tests, which was then counterbalanced across the participants using a Latin-square design.

#### Visual attention network test (vANT)

**Figure 1:**
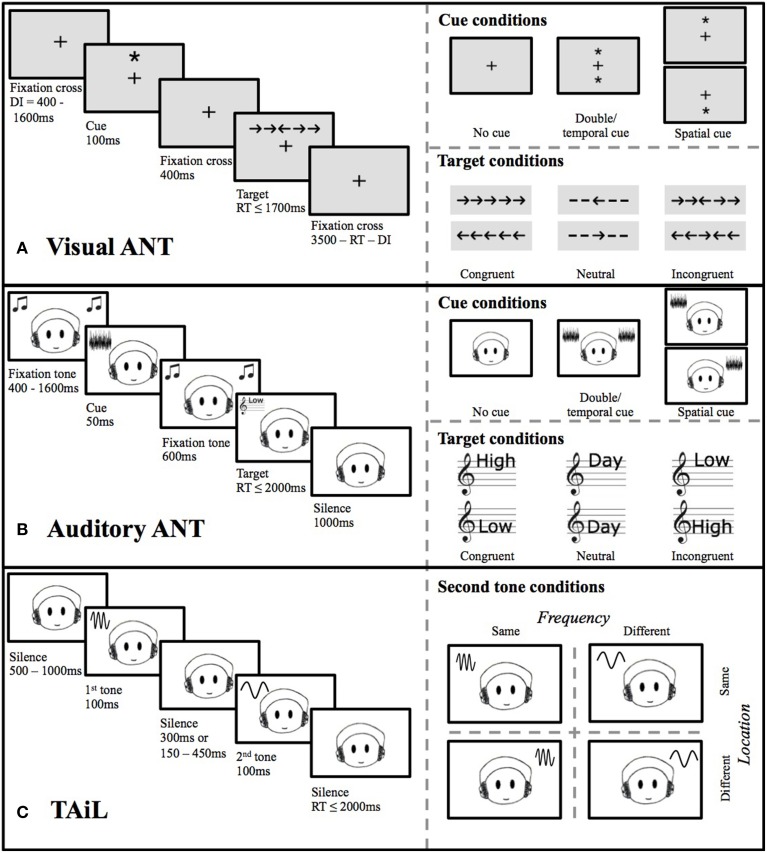
**Trial paradigm illustrations, including cue and target conditions, for the (A) visual ANT, (B) auditory ANT, and (C) TAiL**.

Participants were first presented with a central fixation cross, followed by a visual temporal or spatial cue (an asterisk), or a blank screen in the no-cue condition, to alert participants that the target would occur soon (Figure 1A). The target stimulus (an arrow pointing left or right) was then presented either below or above the fixation cross, alone, or with conflicting/congruent flankers. The participants' task was to indicate via a button press (far left or far right) the direction the target arrow was pointing (task-relevant information), regardless of the flanker arrows (task-irrelevant information). At the seated distance of 65 cm from the screen, the stimuli spanned between 0.5 and 3° visual angle (for a single arrow/line or arrow with flankers, respectively), as described by Fan et al. (2002).

The test consisted of two blocks of 144 trials where all cue types and flanker conditions were randomized within the blocks (4 cue conditions × 2 target locations × 2 target directions × 3 flanker conditions × 3 repetitions). Prior to the first block, participants were provided with verbal instructions and eight practice trials with visual accuracy feedback. RTs from correct trials only were used in the analysis.

Measures of *alerting, orienting* of attention, and *conflict resolution* were calculated from different combinations of cue and flanker trials (see Table 1). *Alerting* was calculated as the difference between trials with a spatial-neutral temporal cue (i.e., a double cue—an asterisk at both possible target locations) and no temporal cue. The participants' *orienting* of attention was calculated as the difference between trials that provided a valid spatial cue (an asterisk at the location of the future target) to those that displayed a spatial-neutral (double) cue. Finally, *conflict resolution* was calculated as the difference between trials where the task-relevant and -irrelevant information (target arrow and flankers, respectively) were incongruent (pointing in different directions) and congruent (pointing in the same direction).

**Table 1:** **Calculations used for outcome measures in the TAiL and the Visual and Auditory ANT, and tasks from the TEA used for measures of ***orienting*** and ***executive control*****.

	***Alerting***	***Orienting***	***Conflict resolution***
vANT	Double cue—no cue	Spatial cue—double cue	Incongruent—congruent
aANT	Double cue—no cue	Spatial cue—double cue	Incongruent—congruent
TEA		Visual elevator task^V^	Telephone task^V^
		Elevator counting with reversal^A^	Elevator counting with distraction^A^
TAiL		Different irrelevant feature—same irrelevant feature	Incongruent—congruent

A *Auditory task*,

V *Visual task*.

#### Auditory attention network test (aANT)

The original stimuli from Roberts et al. (2006) were used. The test set-up is very similar to that of the vANT in that temporal and spatial cues are used, but these cues are auditory tones rather than visual stimuli (Figure 1B). The participant's task was to indicate via a button press whether the speaker's voice was high or low in pitch, whilst ignoring the semantic content (the spoken word “high,” “low” or “day”; an auditory Stroop task). As in the vANT the test consisted of two blocks of 144 trials where all cue types and flanker conditions were randomized within the blocks. Prior to the first block, participants were provided with verbal instructions and 24 practice trials with visual accuracy feedback. RTs from correct trials only were used in the analysis.

The measures of *alerting, orienting* of attention, and *conflict resolution* were calculated as in the vANT (Table 1). *Alerting* was calculated as the RT difference between trials with a spatial-neutral temporal auditory cue (i.e., a double cue—statistically independent noise in each ear) compared to no temporal cue. *Orienting* of attention was calculated as the difference between trials with valid spatial cues (noise in the ear of the future target) and those with a spatial-neutral (double) auditory cue. Finally, *conflict resolution* was calculated as the difference between trials where the task-relevant and -irrelevant information was incongruent (i.e., the word “low” spoken in a high pitch and *vice versa*) and congruent (with matching word and pitch).

#### Test of everyday attention (TEA)

Four subtests of TEA were used to extract measures of orienting of attention and conflict resolution involving auditory and visual stimuli (Table 1). These subtests are described in detail in Robertson et al. (1996), and we present only a short description below.

The visual elevator task presents the participant with a series of pictures and rules to work out what floor an imaginary elevator is on. This subtest has been shown to correlate strongly with classic psychological tasks requiring the participant to switch attention to relevant stimuli (e.g., The Wisconsin Card Sorting Test), therefore providing a visual *orienting* of attention measure (Robertson et al., 1994, 1996). This test was repeated in the auditory domain by the elevator counting with reversal subtest (Robertson et al., 1994, 1996) where the participant counted medium-pitched tones using high and low tones to instruct them when to count the imaginary elevator up and down, respectively. In the Telephone Search task participants visually searched for matching symbols in a “telephone directory” whilst ignoring non-matching symbols. This measure has been shown to be highly correlated with the Stroop task (Robertson et al., 1996; Bate et al., 2001), and so provides a measure of visual *conflict resolution*. Finally the auditory elevator subtest with distraction was used as an auditory *conflict resolution* task where the participant had to count the low tones but ignore the high tones to work out what floor the imaginary elevator was on. The TEA subtests were presented and ordered as described in the TEA manual (Robertson et al., 1996).

Age -normative comparative standards were used to calculate a standardized score for each subtest using subtest-specific look-up tables in the TEA Manual (Robertson et al., 1996). In addition, an individual standard score was used as a *general attention* measure, calculated as formulated by Crawford et al. (1997).

#### Test of attention in listening (TAiL)

Participants heard two successive pure tones that were either same or different in frequency and/or spatial location (ear of presentation), with a roved (150–450 ms) or fixed (300 ms) inter-stimulus interval (ISI) (Figure 1C). Tones were 1 kHz sinusoids of 100–300 ms duration, gated on/off by 10-ms cos ramps and were chosen from the range 476.2–6187.5 Hz, with tone pairs at least 2.1 equivalent rectangular bandwidths (ERBs) (~4 semitones) apart, to be clearly distinguishable. Two tasks were administered, differing only the feature participants had to attend: (1) attend-frequency: participants had to decide whether the two tones had the same or different frequencies while ignoring their location; and (2) attend-location: participants had to decide whether the two tones were presented to the same or different ears whilst ignoring their frequencies. Each task was repeated twice, once with a fixed ISI and once with a roved ISI, with 40 trials per task providing a total of 160 trials per subject. The order of the blocks was counterbalanced using a Latin square design across participants. Each block was preceded by five practice trials, accompanied by on-screen instructions. No feedback on performance was provided. Reaction times (RTs) from correct trials were used in the analysis.

Five outcome measures were calculated from the RT data: *alerting*, which uses both attend-frequency and attend-location tasks, and *involuntary orienting* and *conflict resolution*, each calculated separately for the attend-frequency and attend-location tasks (see Table 1). *Alerting* was calculated as the RT difference between the roved- and fixed-ISI blocks regardless of the attended feature. *The involuntary orienting* measure was calculated as the difference between trials where the task-irrelevant information was the same and where it was different (e.g., attend-frequency trials when the location of the two tones was different minus trials when the location was the same). As in a classic flanker task (Eriksen and Eriksen, 1974), the *conflict resolution* measure was calculated as the difference between trials where the task-relevant and task-irrelevant information were congruent and incongruent (i.e., trials where the location and the frequency were both the same or both different minus trials where either the location or the frequency was different, and the other the same).

### Statistical analysis

One participant was excluded from analysis because of chance performance on one of the TAiL tasks. The remaining participants (*n* = 47) completed all four attention tests.

The RT difference measures of *alerting, orienting*, and *conflict resolution* were assessed for the vANT, aANT using one-sample *t*-tests (test value of 0). The *alerting* measure from the TAiL was assessed using a paired-sample *t*-test comparing roving and fixed-gap tasks. *Involuntary orienting* and *conflict resolution* for the TAiL were assessed using two repeated-measures ANOVAs (one per task-condition) with the task-relevant and task-irrelevant dimension as repeated measures. Involuntary orienting was the main effect of the task-irrelevant dimension, and conflict resolution was the interaction between the relevant and irrelevant dimensions. Only significant measures were included in the factor analysis.

For the factor analysis, the RT difference measures from the vANT, aANT, and the TAiL and the standard scores from the TEA were converted to Z-scores because the raw measures were on different scales. The exploratory principle component analysis (PCA) was carried out using R (R Core Team, 2014) with an oblimin rotation, which allows for both orthogonal and correlated variables. The oblimin rotation provided a well-defined factor structure, with items with factor loadings greater than 0.40 considered appropriate for inclusion in a factor (Fields, 2005).

## Results

### Attention tests

#### vANT

Paired *t*-tests between temporally cued and un-cued trials showed significant alerting [*t*_(47)_ = 4.93, *p* < 0.001]. A comparison of trials with informative spatial and non-informative cues showed significant orienting [*t*_(47)_ = 7.22, *p* < 0.001], and a comparison of congruent and incongruent flankers showed significant conflict resolution [*t*_(47)_ = 14.7, *p* < 0.001]. All three measures were entered into the factor analysis.

#### aANT

Neither alerting (advantage of a temporal cue) nor orienting (advantage of a valid spatial cue) were significant [alerting: *t*_(47)_ = 0.22, *p* = 0.83; orienting: *t*_(47)_ = 0.96, *p* = 0.34]. Only conflict resolution between semantic content and pitch was significant [*t*_(47)_ = 4.13, *p* < 0.001].

#### TEA

Standard scores (mean, range, and SD) as well as population-comparative percentiles for the TEA subtests used in this study are reported in Table 2.

**Table 2:** **Means (M), standard deviations (SD) and mean percentile of the TEA subtests**.

**TEA subtest**	**M (range)**	**SD**	**M Percentile**
*Orienting*^V^ Visual elevator task	12.1 (7–14)	2.0	70.26
*Orienting*^A^ Elevator task with reversal	11.1 (6–13)	2.2	57.86
*Conflict resolution*^V^ Telephone task	8.7 (6–13)	3.4	27.69
*Conflict resolution*^A^ Elevator task with distraction	11.2 (6–13)	2.6	59.12

A *Auditory task*,

V *Visual task*.

#### TAiL

There was no significant alerting effect when tasks with fixed ISI were compared to roved ISI [paired *t*-test: *t*_(46)_ = 0.50, *p* = 0.62]. The involuntary orienting and conflict resolution measures were examined using a 2 × 2 × 2 repeated measures ANOVA (frequency: same, different; location: same, different; task condition: attend-frequency, attend-location). Both measures were significant in both the attend-frequency and attend-location tasks. Involuntary orienting to the irrelevant feature was significant in both the attend-frequency task [*F*_(1, 46)_ = 26.0, *p* < 0.001, $\eta_{p}^{2}=0.37$] and the attend-location task [*F*_(1, 46)_ = 12.1, *p* = 0.001, $\eta_{p}^{2}=0.21$]. Conflict resolution, the difference between congruent and incongruent trials, was also significant in both the attend-frequency [*F*_(1, 46)_ = 14.7, *p* < 0.001, $\eta_{p}^{2}\text{= 0.24}$] and attend-location [*F*_(1, 46)_ = 20.4, *p* < 0.001, $\eta_{p}^{2}\text{= 0.30}$] tasks.

### Exploratory factor analysis

The following 12 measures were included in the factor analysis: *orienting* and *conflict resolution* from the vANT; *conflict resolution* from the aANT; *orienting* and *conflict resolution* using auditory and visual stimuli, and *general attention* from the TEA; and the *involuntary orienting* and *conflict resolution* from both the attend-frequency and attend-location tasks of the TAiL. *Alerting* measures were not included because it was only significant in the vANT, and could therefore not be used to determine modality-specificity/generality in this model.

The factorability of these 12 items was examined. Eight of the 12 items correlated at least 0.30 with at least one other item; Bartlett's test of Sphericity was significant [$\chi_{\left(66\right)}^{2}=290.42,\;p<0.001$]; and the Kaiser-Meyler-Olkin measure of sampling adequacy was 0.57, over the minimum recommendation of 0.50, (Fields, 2005). However, the communality of the visual *conflict resolution* measure from the TEA (the Telephone Search subtest) was low at 0.43, suggesting that this variable did not share common variance with the other items. We therefore proceeded to exclude this item and reexamined the factorability of the remaining 11 items. Eight of the 11 items correlated at least 0.30 with at least one other item. Bartlett's test of Sphericity was significant [$\chi_{\left(55\right)}^{2}=239.65,\;p<0.001$]; and the Kaiser-Meyler-Olkin measure of sampling adequacy was 0.61. All communalities were above 0.50 (see Table 3).

**Table 3:** **Factor analysis loadings with oblimin rotation**.

**Attention measure task**	**General attention**	**Spatial orienting**	**Auditory attention**	**Spatial conflict**	**Communality**
*General attention*^A^^V^ TEA	**0.918**	0.052	−0.002	−0.059	0.861
*Orienting*^A^ Lift reversal—TEA	**0.901**	0.162	0.068	−0.076	0.862
*Conflict resolution*^A^ Lift distraction—TEA	**0.836**	−0.238	0.146	0.015	0.749
*Orienting*^V^ Visual elevator—TEA	**0.627**	0.283	−0.305	0.100	0.665
*Orienting*^V^ vANT	0.253	**0.746**	0.112	0.011	0.643
*Involuntary orienting*^A^ Attend-location TAiL	0.042	−**0.712**	0.162	0.044	0.531
*Involuntary orienting*^A^ Attend-frequency TAiL	0.216	0.077	**0.846**	0.110	0.759
*Conflict resolution*^A^ Attend-frequency TAiL	−0.307	0.288	**0.576**	−0.274	0.586
*Conflict resolution*^V^ vANT	0.089	−0.212	−0.047	**0.770**	0.643
*Conflict resolution*^A^ aANT	−0.321	0.219	0.071	**0.688**	0.662
*Conflict resolution*^A^ Attend-location TAiL	−0.106	0.287	0.364	**0.584**	0.579
**Proportion variance**	28.0	13.6	13.0	13.0	67.6

A *Auditory task*,

V *Visual task*.

*Bold values indicate the items that load onto that column's component*.

Given the results from these initial tests, principle components analysis was conducted with all 11 measures, as the aim of the study was to explore the underlying relationships of the modalities of different attention tests.

Principal components analysis indicated the presence of four factors with eigenvalues greater than Kaiser's criterion of 1 (Kaiser, 1960). This was supported by parallel analysis and Cattell's scree plot test (Cattell, 1966), with the four factors explaining 67.6% of the cumulative proportion of variance.

#### Factor loading

**Figure 2:**
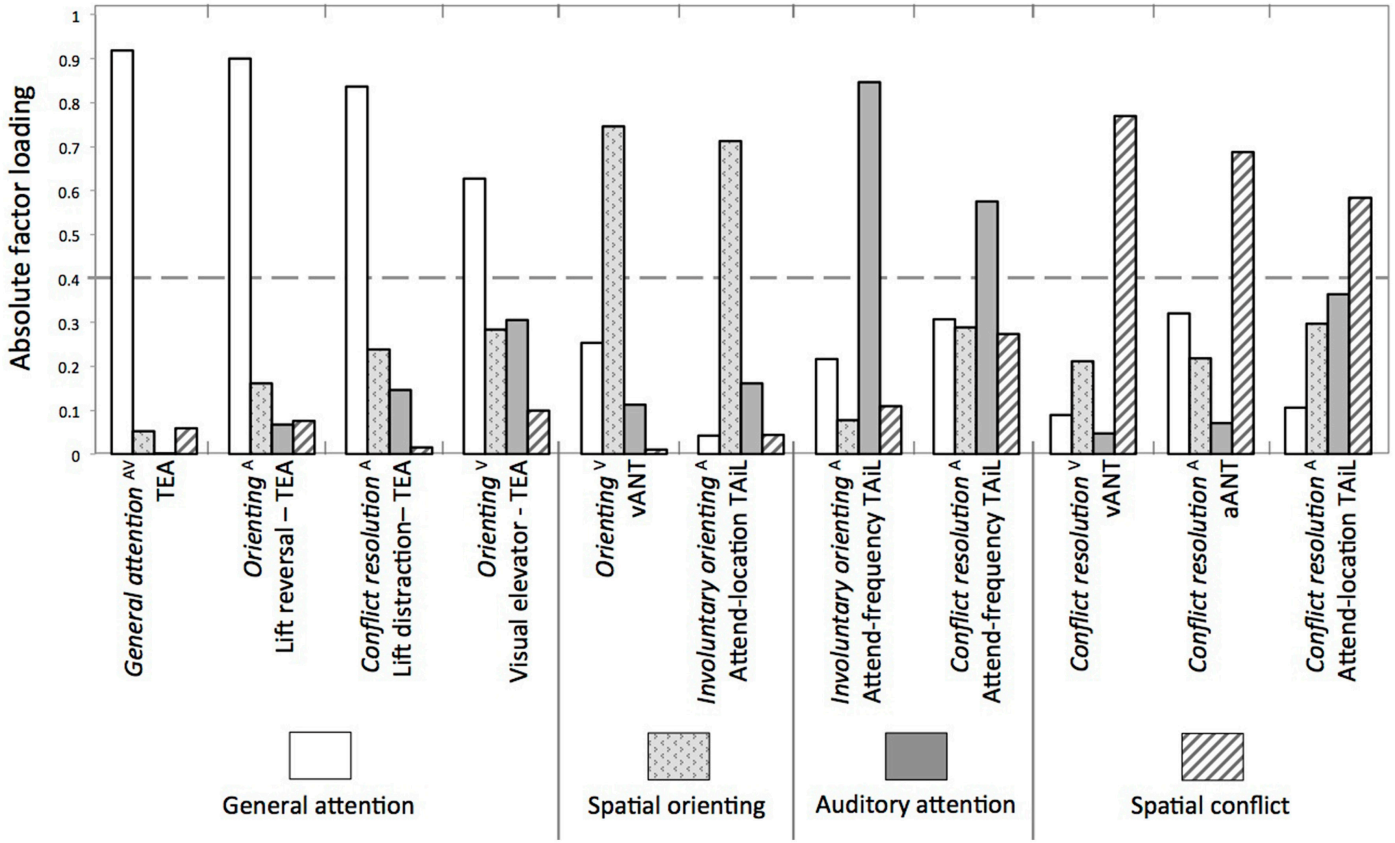
**A visual representation of Table 3—the absolute factor analysis loadings with oblimin rotation**. Dotted horizontal line indicates the cutoff of item loadings considered for each factor (i.e., 0.4). ^A^auditory task, ^V^visual task.

Principal components analysis yielded a 4-component solution. The factor loading matrix is presented in Table 3, and illustrated in Figure 2. All four TEA measures loaded onto the first component: general attention—consisting of both auditory and visual subtest scores; orienting from the visual elevator task score; and the two conflict resolution measures from the two auditory elevator counting subtests (reversal and distraction). We named this component “general attention.”

The second component consisted of the *orienting* measure from the vANT and the *involuntary orienting* measure from the TAiL attend-location task. Both of these items are spatially based *orienting* measures from tasks requiring a direction based decision (i.e., left/right) covering both audition and vision. Hence, we suggest this is a supramodal “spatial orienting” component.

Two items loaded onto the third component: both the *involuntary orienting* and *conflict resolution* from the TAiL attend-frequency task. This component appears to be auditory-specific and was labeled “auditory attention.”

The final component consisted of three *conflict resolution* measures from the vANT, aANT, and the TAiL attend-location task. We suggest this component is also supramodal, and labeled it “spatial conflict” as each task involved a directional decision (i.e., left/right or high/low).

## Discussion

This study explored the underlying constructs of selective attention based on the Attention Network framework (Posner and Petersen, 1990) and their modality specificity or generality. Measures of selective attention from the auditory and visual tests used in this study did separate into auditory-specific and supramodal components, but the expected visual-specific component(s) were not identified. Contrary to our expectations, modality-specificity, and generality did not correspond to the attention networks. The auditory-specific component consisted of both orienting to and conflict resolution of the non-spatial feature that defined the auditory object, namely pitch, whereas all of the supramodal components were related to spatial aspects of either sound or visual stimuli (e.g., the direction of the target arrow or the ear of sound presentation). Orienting to location and resolving spatial conflict from both modalities loaded onto the same orienting and conflict resolution components, respectively. Only selectively attending to non-spatial auditory features loaded onto a separate component. We suggest that there was no equivalent visual-specific component because the visual tests used did not contain task-relevant non-spatial features.

### “What” and “where” pathways in vision and audition

Although the results do not support our original hypothesis regarding the specificity and generality of attention networks, the resulting division of attention measures into spatial and non-spatial principle components reflect the theory of dual-pathways (Mishkin et al., 1983; Goodale and Milner, 1992). This theory posits that sensory stimuli are processed in two separable pathways: a “where” pathway processing spatial information and a “what” pathways processing non-spatial object-related information. This theory is supported by numerous imaging studies in both human (e.g., Haxby et al., 1991; James et al., 2003; Zachariou et al., 2013) and animal (e.g., Desimone et al., 1985; Baizer et al., 1991; Felleman and Van Essen, 1991) vision, corresponding to anatomically separate ventral and dorsal streams processing the “what” and “where” features of a visual object, respectively. A similar dual-pathway theory has been proposed for audition (see Rauschecker and Tian, 2000; Arnott et al., 2004).

Based on the anatomical evidence, this theory suggests that whilst processing the spatial features of an object is supramodal, the non-spatial features are processed in a modality-specific fashion (Driver and Spence, 1994, 1998; Turatto et al., 2002). Imaging studies comparing the cortical activation elicited by spatial and non-spatial feature processing in the visual and auditory domains have suggested that selective attention for spatial auditory features engage cortical circuitry similar to that engaged in visual spatial selective attention (e.g., Krumbholz et al., 2009; Smith et al., 2010). This network is referred to as the dorsal stream, consisting of a superior parietal and frontal network of regions that is activated by tasks requiring spatial orienting or conflict resolution in either visual (Giesbrecht et al., 2003; Slagter et al., 2007) or auditory (Ahveninen et al., 2006; Hill and Miller, 2010; Lee et al., 2013) modality. Our behavioral results mirror these imaging studies by showing that the spatial-based task measures loaded onto the same two components—one for orienting of attention and one for conflict resolution.

Imaging evidence suggests that orienting to non-spatial object features leads to activation in separate, modality-specific sub-networks along ventral regions. In audition it includes areas in the non-primary auditory cortex in the temporal lobe (e.g., the superior temporal sulcus; (Arnott and Alain, 2011) activated by non-spatial auditory object features (e.g., pitch) while in vision it includes extrastriatal visual areas in the inferior temporal gyrus (Giesbrecht et al., 2003) activated by non-spatial object features (e.g., color). Our behavioral results suggest a divide between the processes involved in attending to auditory spatial and non-spatial features. However, the current experiment was not designed to contrast spatial and non-spatial visual attention, and further study will be required to confirm this distinction.

### Visual and auditory alerting measures

Some discussion of each of the three selective attention networks is warranted. It is interesting that we found no evidence of alerting in either auditory test compared to the visual ANT. In the TAiL there was no significant RT advantage for the fixed vs. roved ISI suggesting that knowing when the second tone would be presented did not lead to faster responses. We also failed to replicate the significant alerting effect Roberts et al. (2006) found in the aANT when comparing RTs in the presence of a temporal cue that indicated when the target will occur, compared to a no-cue condition. Despite the differences between the types of decision required by the two tasks—discrimination in the TAiL and identification in the aANT—the main question here is why there is no apparent auditory alerting, whereas a robust visual alerting effect found in the vANT.

Auditory detection is much more rapid than visual detection; the time from stimulus onset to arrival at primary sensory cortex is considerably shorter in the auditory modality (for a review see Hillyard, 1993). We speculate that the latency advantage conferred by knowing exactly when an auditory “target” will occur may be too small to detect with any precision using the RT measures of the aANT and the TAiL. Alternatively, the lack of an alerting effect in one modality which is present in the other might in itself indicate that alerting is modality specific. Spagna et al. (2015) found uncorrelated auditory and visual alerting when using a double cue compared to no cue. Since the TAiL does not include a no-cue condition, we do not have an equivalent measure. Thus, we cannot conclude whether alerting is a sensory-specific or supramodal function based on this study.

### Orienting to stimuli and test relevance

The RT tests used in this study tapped into two different types of orienting: orienting to a non-spatial feature of an object (frequency in the TAiL attend-frequency task), compared to orienting to the spatial location of an auditory (TAiL attend-location task) or visual (vANT) object. In the vANT, the location cue was relevant to the task, as knowing where the target will appear on the screen allows covert attention to be moved to that location, reducing target processing time. The TAiL, on the other hand, does not measure the benefit afforded by a cue that orients attention to the task-relevant information, but rather the resistance to distraction by irrelevant information. When the relevant information is spatial (attend-location), it is a measure of how well participants can orient to location and ignore other stimulus features. Indeed, this measure loaded on a spatial orienting component together with the vANT *orienting* measure. With both a visual and an auditory measure loading on this component, spatial orienting appears to be a supramodal function.

Unlike the vANT, the spatial location cue in the aANT (ear of presentation) is irrelevant to the required decision about the pitch of the word. It is possible that knowing the future location of an auditory object does not help with identifying its features (McDonald and Ward, 1999). Moreover, the well-established right-ear advantage for speech (for a review see Hugdahl, 2011) may have confounded any putative advantage of a spatial orienting cue, resulting in no significant orienting effect in the aANT here or in the Roberts et al. (2006) study. Spagna et al. (2015), also showed a lack of spatial orienting effect in a non-verbal auditory ANT. In their task, the decision on target pitch was unaffected by a spatial cue.

By comparison, the attend-frequency task of the TAiL required orienting to a non-spatial stimulus feature, unlike the vANT which measured only spatial orienting. It therefore follows that this measure did not load on the “spatial orienting” component. Since the aANT did not have a measure of non-spatial orienting, we can only suggest that orienting to non-spatial features is modality-specific. This conclusion is supported by Spagna et al. (2015), who showed a significant orienting effect to tone pitch in a non-spatial version of the auditory ANT, which was uncorrelated with the vANT.

Although it has been suggested that the TEA visual elevator subtest taps into the orienting network (Robertson et al., 1994, 1996), it did not load on either the spatial orienting component or a sensory-specific orienting component. This is not surprising as it is a rule-based attention switching task, and the modality of the cue is irrelevant—only the rule matters to task performance.

### Conflict resolution and the role of semantics

The TAiL non-spatial *conflict resolution* loaded on the same auditory-specific factor as the non-spatial *involuntary orienting* measure, also from the attend-frequency task. It is perhaps surprising that the aANT *conflict resolution* measure did not load onto the auditory-specific component, since this task's Stroop conflict was between the semantic content of the word and its pitch—not overtly a spatial conflict. Our findings echo the correlation between the aANT and vANT conflict resolution found in the Roberts et al. (2006) study. It is possible these two loaded onto a spatial conflict resolution measure because the word meanings in the aANT were spatial (“high,” “low”). Therefore, the TAiL spatial *conflict resolution* (attend-location), the aANT *conflict resolution* and the vANT *conflict resolution*, also spatial (left and right arrow flankers), loaded onto the same factor. Thus, like spatial orienting, spatial conflict resolution appears to be supramodal.

Non-spatial conflict resolution requires more investigation. Firstly, Spagna et al. (2015) found two auditory non-spatial conflict resolution measures (to pitch and duration) to be moderately correlated with the vANT (but only for the Spearman correlations). In contrast, the TAiL non-spatial, pitch-based conflict resolution did not load onto the same factor as the vANT. Secondly, the current study did not include a non-spatial visual conflict resolution measure. We therefore cannot categorically conclude that non-spatial conflict resolution is modality-specific.

### The TEA and working memory

Although the TEA is purportedly based on the Attention Network framework (Robertson et al., 1994), none of its measures loaded onto components with any other tests of selective attention used here. Whilst the subtests of the TEA were designed to be ecologically valid, they are rule-based, the cues (both auditory and visual) used to direct attention have no meaning in themselves, but rather direct attention to a rule that needs to be remembered and used correctly. In fact, factor analysis studies have shown the visual elevator task and auditory elevator tasks, both with reversal and distraction, to load onto the same factor as working memory tasks such as the backwards digit span and Paced Auditory Serial Addition Test (PASAT, Gronwall and Wrightson, 1974: where the listener adds an auditory number to the previously heard number) (Robertson et al., 1996; Bate et al., 2001). We suggest that this may be a separable component whose underlying construct is based in working memory.

## Conclusion

Our results suggest that the networks of selective attention—alerting, orienting and conflict resolution—are not in themselves modality-specific or supramodal. The exploratory factor analysis suggests that attending to spatial stimulus features is supramodal. However, we cannot make firm conclusions regarding attention to non-spatial features. Future studies should include measures of non-spatial visual selective attention in a confirmatory factor analysis.

## Author contributions

HJS and SA designed the study. HJS conducted the study and analyzed the data. HJS and SA wrote the manuscript.

### Conflict of interest statement

The authors declare that the research was conducted in the absence of any commercial or financial relationships that could be construed as a potential conflict of interest.
